# Evaluation of Land Comprehensive Carrying Capacity and Spatio-Temporal Analysis of the Harbin-Changchun Urban Agglomeration

**DOI:** 10.3390/ijerph18020521

**Published:** 2021-01-10

**Authors:** Yang Tang, Yongbo Yuan, Qingyu Zhong

**Affiliations:** 1 Faculty of Infrastructure Engineering, Dalian University of Technology, Dalian 116024, China; tangyang@mail.dlut.edu.cn; 2 Dalian Municipal Facilities Construction Co. Ltd., Dalian 116011, China; garyzhong2021@126.com

**Keywords:** land comprehensive carrying capacity, comprehensive index evaluation, spatial-temporal differentiation, Harbin-Changchun urban agglomeration

## Abstract

Land comprehensive carrying capacity (LCCC) reflects the limits of urban development that the land ground in the spatial area can bear under the constraints of society, economy, resources, and environment. An accurate and objective evaluation of LCCC is of great significance to the rational planning of urban space. Using the entropy method to obtain the weights of evaluation indexes, this study constructed an index system composed of four subsystems, i.e., urban construction (UC), social economy (SE), industry development (ID), and urban ecology (UE). Furthermore, calculating the index of 11 cities for the Harbin-Changchun urban agglomeration (HCUA) to analyze the influence of LCCC from diverse dimensions for the period 2004–2015. Lastly, the spatial and temporal differentiation characteristics between the neighboring units of LCCC were visualized through global and local spatial analysis. The results infer some novel findings as follows. (1) The overall tendency of the LCCC of the HCUA has gradually increased over the time window with the coordination of each subsystem. The urban ecological subsystem shows the highest rate of contribution and the social economy subsystem has the largest increase. Urban construction and industry development have a lower number of hot spot cities and lower clustering characteristics than social economy and urban ecology. (2) The core cities of the HCUA, Changchun and Harbin, demonstrate the reverse trend from 2010 to 2015, which reveals the states of excellent and good carrying capacity, respectively. In contrast, four cities are at the inferior state, and three cities are poor. Prioritizing the promotion of the industry development subsystem should be considered for these seven cities. (3) There is some spatial variation of LCCC in the HCUA, which shows the characteristic of “gradually decreasing from the core city to the surrounding area”. Changchun and Jilin are high–high clustering areas that drive Siping from a cold spot to a hot spot. Focusing on the development of secondary growth pole cities, Jilin and Songyuan are forming complementary and mutual reinforcement with the core cities, which has a positive significance in promoting the sustainable development of the regional space of urban agglomeration.

## 1. Introduction

### 1.1. Background

The city is the space carrier of social economy, undertaking the role of constructive development activities. Urban agglomeration is an inevitable result of the development of urbanization to an advanced stage [1,2]. Looking in retrospect at the development procedure of the major advanced nations worldwide, urban agglomeration plays a prominent role in participating in international competition, optimizing function layout, and implementing public policies. Since 2014, the Chinese government has formulated a series of development strategies including the National New Urbanization Plan (2014–2020), in which urban agglomeration has become the main unit to promote new urbanization. Meanwhile, the Harbin-Changchun urban agglomeration (HCUA) has been officially listed as the locally critical focus region of key exploitation in China [3].

Land resources are the foundation and premise of human survival and development. Due to high-intensity social economic activities and rapid urbanization, the overloaded condition of global resources has started to emerge [4,5,6]. According to the United Nations Department of Economic and Social Affairs Outlook Report on the World Population in 2019, the world’s population will reach 9.7 billion by 2050, which will result in land resources that are no longer sufficient for population growth and urban development [7]. Since 1978, the urbanization rate in China has increased by nearly 41%, which has led to a mismatch between the intensity of land exploitation and the carrying capacity of resources in some areas. The phenomenon of unbalanced land development is becoming extremely severe. Therefore, the shortage of land resources and the inefficient use of land resources have become the main obstacles that affect and restrict urban development [8,9]. In addition, China has successively proposed the Major Function Oriented Zoning (MFOZ) and the National Land Planning Outline [10], which guide the orientation of the eco-friendly growth mode of land space and the elevation of land use efficiency. Motivated by these stylized facts, we believed that a spatial and temporal evaluation of land carrying capacity from a particular perspective would provide a meaningful study.

### 1.2. Literature Review

Carrying capacity was originally used to measure whether the key load of an object was damaged. It was first cited by the field of ecology and defined as follows: carrying capacity is the threshold value of the maximum human activity capacity under certain conditions [11,12]. For previous studies of the theoretical connotation of land carrying capacity, foreign research started earlier and laid the foundation for future research, which included the single-factor carrying capacity and the environmental carrying capacity, with the former focusing on the human–food relationship [13,14]. The latter focuses on the level of human activities that regional land resources can carry under natural resource constraints [15,16]. In further research, land carrying capacity was extended to include not only ecological factors but also social factors, such as population, capital, and infrastructure, associated with the development of urban areas, of both natural and anthropogenic systems [17]. The latest research indicates that land comprehensive carrying capacity (LCCC) will become the scientific basis and core indicator for future urban spatial territorial planning and sustainable development [18].

The development of land carrying capacity models have been promoted by the rise of comprehensive research theory and method. LCCC is particularly important in the study of carrying capacity. Most of the models identify the factors of land resource carrying capacity, establish an evaluation system, determine the weighting of each factor, and complete the evaluation by various methods [19,20]. Several models are used in the evaluation of land carrying capacity, such as the ecological footprint method [21,22], the system dynamics model [23,24], and the comprehensive evaluation method [25]. Nakajima assessed the land use of Ibiúna County through the ecological footprint method, which proved the necessity of changing the county’s economic structure. The method was adapted to obtain an alternate carrying capacity for the county that was conducive to the sustainable development of the country [26]. Qian et al. constructed an improved ecological footprint model for Xiamen City and established a methodology for land carrying capacity criteria, which showed that land carrying capacity was reduced by arable land reduction and energy consumption. Therefore, the protection of land types such as cultivated land or forest had a positive impact on the construction of low-carbon cities [27]. The system dynamics model explores the causal chains that affect land carrying capacity, and quantitatively analyzes the internal mechanism between the structure and function [28,29]. Aspinall established a land system dynamics framework to explore the land use under different conditions. The model considered capital funds and flows as a set of driving subsystems, illustrating how driving subsystems were influenced by the linkages between human processes and the environmental system, achieving the dynamic assessment of land cover [30]. The comprehensive evaluation method constructs a system of multiple index layers, based on the fact that each index layer such as resource, environmental, economic, social, and other factors are additive [31,32], so as to achieve the quantitative evaluation of carrying capacity.

At present, the study of the LCCC of China’s national-level urban agglomerations has become of great importance. The geographical proximity of urban agglomerations is conducive to resource allocation. In addition, the land resource efficiency of urban agglomerations is higher than that of non-urban agglomerations, which are positively related to the degree of spatial agglomeration [33]. Liu assessed the carrying capacity of urban agglomerations in the Yangtze River Delta and determined the key limiting factors for land resources. The study proved that there was a spatial and temporal difference in carrying capacity with a growing trend [34]. Li predicted the land carrying capacity of the urban agglomeration in the Pearl River Delta and simulated the land use pattern under different scenarios to propose an optimal land use pattern [35]. Wang evaluated the land use of the Shandong Peninsula urban agglomeration by establishing a discriminant model with a three-dimensional matrix of development intensity, carrying capacity, and utilization efficiency. Key development zones, stable development zones, and restricted development zones were determined by thresholds [36]. In addition, some scholars explored the spatial pattern and changing characteristics of the land resource carrying capacity of urban agglomerations [37,38].

Some limitations of existing studies on LCCC include the following. (1) Most studies about spatial changes in land carrying capacity have focused on land use and cover changes. There has been little discussion of land carrying capacity changes between each spatial unit and its neighbors. Hence, the influence of the regional geographic distribution structure in land carrying capacity has been neglected. (2) There are few LCCC studies on China’s regional urban agglomerations. Research mainly aims at the national level. Regional urban agglomerations are the key urbanized areas of the country to drive regional development, with increasing natural resources, social resources, economic output, and population, which provide a guarantee for the development of national-level urban agglomerations. However, the lack of LCCC studies on regional urban agglomerations is not conducive to the establishment of a unified early warning mechanism for land carrying capacity.

### 1.3. Research Objectives and Innovations

To sum up, we constructed an LCCC evaluation index system, which included four dimensions of urban construction activities and resource environment. By evaluating the LCCC of 11 cities for the HCUA, we are able to determine the gradients of LCCC and the main factors affecting each gradient. Furthermore, we explored the spatial differentiation and variation between neighboring units of the LCCC in the HCUA. This study adds to existing research in the following ways. (1) The evaluation dimension of the LCCC index was enriched from the following two aspects. On the one hand, when evaluating land productivity, we creatively took both urban construction activities and industrial development into consideration. On the other hand, we selected an index based on the Technical Requirements for Land Resources and the Environmental Carrying Capacity Evaluation, so that the evaluation system was objective and consistent with domestic land resource use and the comparability of evaluation results was improved. (2) The distribution and spatial characteristics of the LCCC were revealed by the spatial analysis under the geographic structure characteristics of the dual-core urban agglomeration. Considering the regional differences between cities, we optimized the system by geographical weight setting of the spatial analysis. This was done to avoid the unity of different cities using the same standard in former studies. (3) This study selected the regional urban agglomeration of HCUA as the research object. The HCUA is representative of regional dual-core urban agglomeration in China. Studying the LCCC of the HCUA can not only support an understanding of the current status, but also provide a reference for the evaluation of LCCC in other dual-core urban agglomerations. The remainder of the study is organized as follows. Section 2 outlines the theoretical implications and the construction of an index system for evaluating LCCC. Section 3 presents the methodology and data. The results analysis and discussion are illustrated in Section 4. We conclude this study in Section 5.

## 2. Theoretical Implications and Construction of an LCCC Index system

### 2.1. Dimensions of the LCCC Evaluation Index System

LCCC refers to the consumption of resources and environment by all human economic activities. Achieving sustainable land use is an important mission of contemporary urban development, which can balance the needs between the present and future generations [39]. It is a process of striking a harmony among economic, social, and environmental factors. In order to achieve this goal, land resources need to maintain various carriers that have the capacity to carry socioeconomic and construction activities, such as environment, transportation, infrastructure, and capital resources. These are considered as components of LCCC (see Table 1).

**Figure 1:**
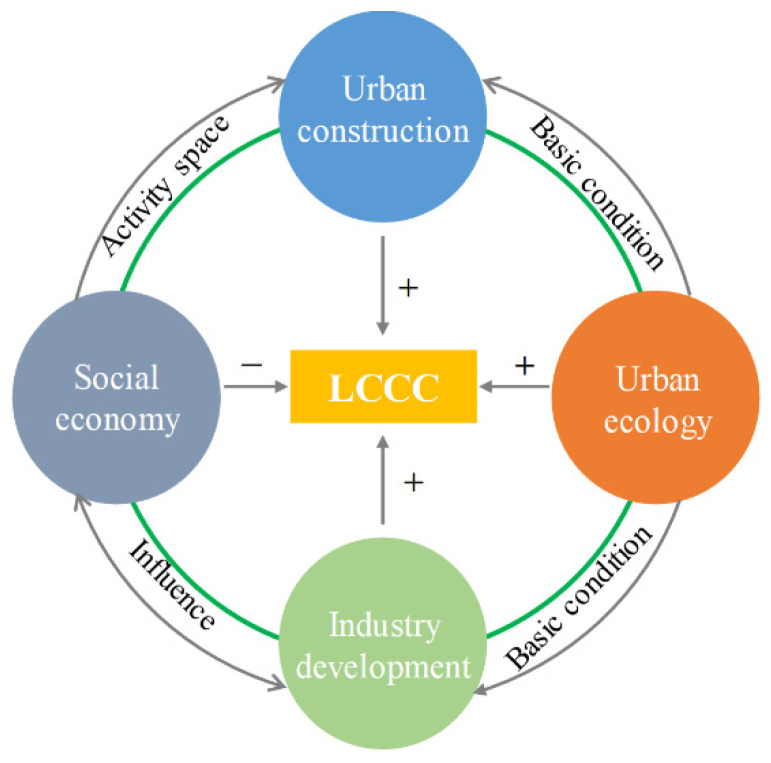
Subsystem interactions of LCCC.

LCCC refers to the consumption of resources and environment by all human economic activities. It can be divided into four subsystems, namely, urban construction (UC), social economy (SE), industry development (ID), and urban ecology (UE). UC provides the necessary space for human activities. UE has a constraining effect on social economy and industry development. This study evaluates the level of LCCC by measuring the combined effect of the four subsystems (Figure 1).

### 2.2. Construction of an LCCC Evaluation Index System

#### 2.2.1. Basis for Index Selection

Index selection follows three principles. First, selecting an index that is generally accepted by existing research studies to improve the hierarchy and rationality. Second, referring to the indexes for land resource evaluation in the “Technical Requirements for the Evaluation of the Environmental Carrying Capacity of Land Resources “proposed by the Ministry of Natural Resources of China, so that the index system is more in line with China’s national conditions [45]. Third, combining the comprehensiveness and timeliness of data to improve the operability of the index system.

To avoid data distortion caused by the population and geographical area of different regions, we used the land average, utilization rate, and percentage to construct the index system. In order to highlight the particularity of the HCUA, the construction of the criterion layer was focused on the urban construction situation and land use level in the northeastern part of China. Social economy indicates the size of the regional economy and the degree of urbanization. Urban ecology supports urban construction and industrial development (Table 2).

#### 2.2.2. Determination of Indicators

(1) Urban construction (UC). An opening area for promoting sustainability with much external contact and internal communication [46]. The specific indicator per capita housing area [18,41,42] reflects the degree of urban land construction and the scale of urban living space. Considering the characteristics of interconnection and openness of urban agglomerations, the construction of roads can accelerate the flow of factors between cities. Hence, the road density [47] index was chosen to reflect the development of construction land. Per unit area infrastructure investment [43,48] describes the intensity of land input and output.
(2) Industry development (ID). Increased social and economic scale is an important factor in urban land expansion. It usually improves the income and living standards of urban residents, resulting in an increase in population size and mobility. Changes in the needs of the population and in production patterns have led to changes in land use [49]. Economic density [18,41,42] refers to the output efficiency that can be created per unit area of land, reflecting the economic development of the city itself as the population changes. Engel’s coefficient [40,50] reflects the level of living of urban residents. Urbanization rate [18,42,43] indicates the scale of population gathering. On the one hand, it reflects the development scale of regional economy. On the other hand, it measures the support of land population carrying to the economic development.
(3) Social economy (SE). The development of the primary and secondary sectors is compatible with the level of input and output efficiency of urban land, which greatly supports the livelihood of the urban population and economic development. According to extensive studies on urban industries, high-quality industrial development land resources are used more efficiently [48,49]. The scale of arable land supply to the population expressed through the per capita farmland area [41,51] indicator, which can properly illustrate the basic support of regional land for industrial development and is a guarantee of arable land for the sustainable use of land resources. For the perspective of output, per unit area industrial output [43,44] reflects the carrying efficiency and use intensity of unit land, which is an important factor in evaluating LCCC. Per capita grain output [42,43,51] is the direct indicator of the carrying capacity of land production scale, that is, the supply capacity to ensure the basic survival needs of the urban population.
(4) Urban ecology (UE). The ecological environment is the basis for supporting the sustainable development of cities [50,52]. As an important strategic area for the revitalization of old industrial bases in the Northeast, the HCUA should attach great importance to the construction of self-cleaning capacity and ecological environment. Resource- and environment-related indexes classified into the urban ecology subsystem, such as the domestic sewage treatment rate [51,52], represent the ecological adaptive capacity of human activities, which deeply reflects the intensity and importance of ecological treatment and environmental protection issues. As an important resource to support productive activities, per capita green area [18,43,51] can reflect the quality of the living environment of urban residents. Green coverage of urban area [52,53] describes the natural condition carrying capacity of the land.

## 3. Data and Methods

### 3.1. Study Area and Data Sources

**Figure 2:**
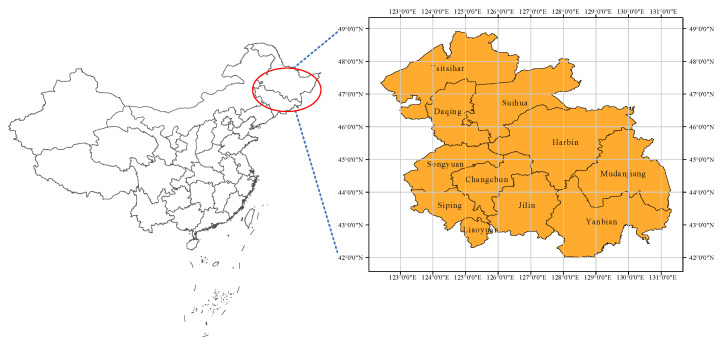
Location and range of the Harbin-Changchun urban agglomeration (HCUA).

The HCUA, which is located between 42°05′ N and 49°38′ N,123°57′ E and 131°36′ E, lies to the north of the longitudinal axis of the Beijing-Harbin Railway in the national two horizontal and three vertical urbanization strategic patterns (Figure 2). Occupying the first position of nine key regional urban agglomerations proposed in China’s Thirteenth Five-Year Plan, the HCUA is the gateway to China’s Northeast Asian export-oriented economic construction.

In terms of geographical structure, the HCUA is a typical dual-core urban agglomeration, covering 263,300 square kilometers of national territorial area, with the capital cities of Harbin and Changchun as its center. It includes Harbin, Daqing, Qiqihar, Suihua, and Mudanjiang of Heilongjiang Province as well as Changchun, Jilin, Siping, Liaoyuan, Songyuan, and Yanbian of Jilin Province.

The study years are from 2004 to 2015. The data were collected according to the index system, which mainly includes statistical yearbooks, statistical bulletins, and environmental status bulletins. The data sources are shown in Table 3.

### 3.2. Assessment Method of the LCCC

#### 3.2.1. Data Standardization

The evaluation of the LCCC index included the following three steps: data standardization, weight determination, and index value estimation.

In the first step, dimensionless standardization was employed to eliminate the influence of different dimensions of the original index data. For the treatment of income indicators (the greater the better), see Equation (1); for the treatment of cost indicators (the smaller the better), see Equation (2).

(1) $$x_{ij}^{*}=\frac{x_{ij}-\min(x_{j})}{\max(x_{j})-\min(x_{j})}\left(i=1,2,\ldots ,t;j=1,2,\ldots ,p\right)$$

(2) $$x_{ij}^{*}=\frac{\max(x_{j})-x_{ij}}{\max(x_{j})-\min(x_{j})}\left(i=1,2,\ldots ,t;j=1,2,\ldots ,p\right)$$

where the subscript *i* denotes the city *i*, and *j* represents each index; *t* and *p* indicate the number of cities and indexes, respectively; max(*x_i_*) and max(*x_j_*) refer to the maximum and minimum values of the index *j* in all urban agglomeration cities. Then, different attributes of the basic index are consistent and comparable.

#### 3.2.2. Weight Determination

In order to improve the objectivity of the carrying capacity evaluation, considering the complexity of urban resource–environment–social system and the uncertainty of the index of the LCCC, the weight of the LCCC index was determined by the entropy weight method [54]. The specific calculations are as follows:

(a) Calculate the entropy value *e_j_* of index *j* with Equation (3).
  (3) $$e_{j}=-\frac{1}{\ln n}\sum_{i=1}^{n}p_{ij}\ln p_{ij}$$
(b) Calculate the difference coefficient *g_j_* of index *j* with Equation (4). The bigger the entropy value, the less important the index.
  (4) $$g_{j}=1-e_{j}$$
(c) Calculate the weight of index *j* with Equation (5), where all the index weights are equal to 1.
  (5) $$w_{j}=\frac{g_{j}}{\sum_{j=1}^{n}g_{j}}$$

#### 3.2.3. LCCC Value Estimation

In this study, we used the multi-objective linear summation method to calculate the LCCC of the HCUA according to the normalized values and the corresponding weights of each index. These are described in Equations (6) and (7), respectively:

(6) $$S_{is}=\sum_{j}^{J}x_{ij}^{*}w_{j}$$

(7) $${\mathrm{LCCC}}_{i}=\sum_{s=1}^{4}S_{is}\;\left(i=1,2,3,\ldots I;j=1,2,3\ldots J\right)$$

where *S_is_* is the carrying capacity of the subsystem and ${\mathrm{LCCC}}_{i}$ is the value of land comprehensive carrying capacity for city *i*.

### 3.3. Global and Local Spatial Autocorrelation

Generally, due to the proximity of the geographical structure of urban agglomerations, the land carrying capacity has a spatial correlation effect and a radiation effect on surrounding units [55]. The ability of the urban land resource system to withstand external disturbance becomes stronger as the regional index increases. Therefore, the spatial autocorrelation method was used to measure and analyze the spatial agglomeration and distribution trends of the HCUA. Global Moran’s I was used to assess the degree of spatial agglomeration of the land comprehensive carrying capacity of the HCUA [56]. The equation is as follows:

(8) $$I=\frac{\sum_{i=1}^{n}\sum_{j=1}^{n}w_{ij}\left(x_{i}-\bar{x}\right)\left(x_{j}-\bar{x}\right)}{\frac{1}{n}\sum_{i=1}^{n}{\left(x_{i}-\bar{x}\right)}^{2}\times \sum_{i=1}^{n}\sum_{j=1}^{n}w_{ij}}$$

where *I* denotes the global Moran’s I statistic of LCCC; $w_{ij}$ is the spatial weight matrix, which means the spatial positions’ relationship between *i* and *j*; $x_{i}$ and $x_{j}$ are the LCCC values in cities *i* and *j*, respectively; $\bar{x}$ is the mean value of LCCC; and *n* is the number of study objects. We selected the most common adjacency method to establish the spatial weight matrix under the geographic features. The range of *I* is (−1,1). When *I* > 0, it indicates a positive correlation in the distribution of land comprehensive carrying capacity. That is, it has spatially aggregated distribution characteristics. When *I* < 0, it indicates a negative correlation in the distribution of land comprehensive carrying capacity, showing spatially dispersed distribution characteristics. When *I* = 0, this is a randomly distributed state.

Local Moran’s I reflects the correlation of LCCC between local areas. The LISA (Local indicators of spatial association) agglomeration map is applied to represent the spatial agglomeration distribution of each city and surrounding cities. There are four types of results, i.e., high–high clustering, low–low clustering, high–low clustering, and low–high clustering. Therefore, local Moran’s I can explore the spatial relationship of the carrying capacity of city clusters in more detail.

## 4. Results and Discussion

### 4.1. Overall Analysis of the LCCC for the HCUA

**Figure 3:**
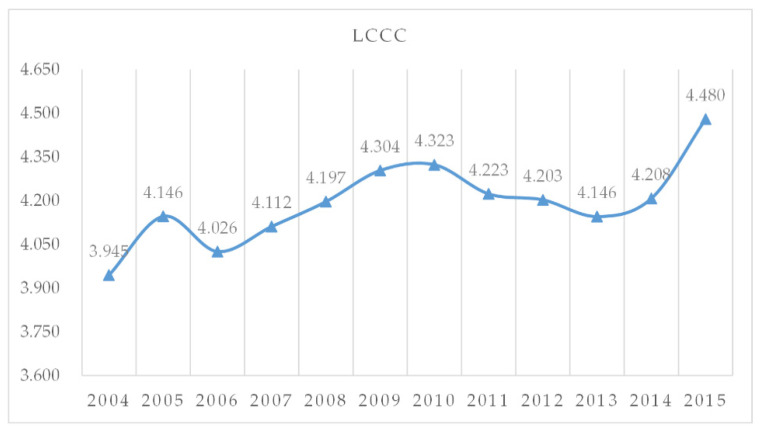
The overall tendency of the LCCC in the HCUA during 2004–2015.

Figure 3 illustrates the overall tendency of the LCCC curve in the scale of the whole HCUA during the period of 2004–2015.The upward trend indicates that sustainable land use is gradually increasing. In addition, fluctuations in the trend curve means that LCCC changes in response to the external environment and policies. Specifically, the first low point of the curve appeared in 2006 when urban infrastructure and public service were in short supply due to the rapid urbanization process, which brought about massive population movements. According to index data, the domestic sewage treatment rate declined rapidly in 2005–2007, indicating that the growth of the population led to a shortage of urban supply capacity. The second low point came in 2013, when the new normal economy brought about a slowdown in GDP growth, forcing resource-based cities in the HCUA to change the way of development [57]. After 2013, a steady upward trend is shown; investment in infrastructure increased, because the trial operation of the Harbin-Dalian high-speed railway accelerated the flow of factors in the urban agglomeration and improved the allocation of resources.

**Figure 4:**
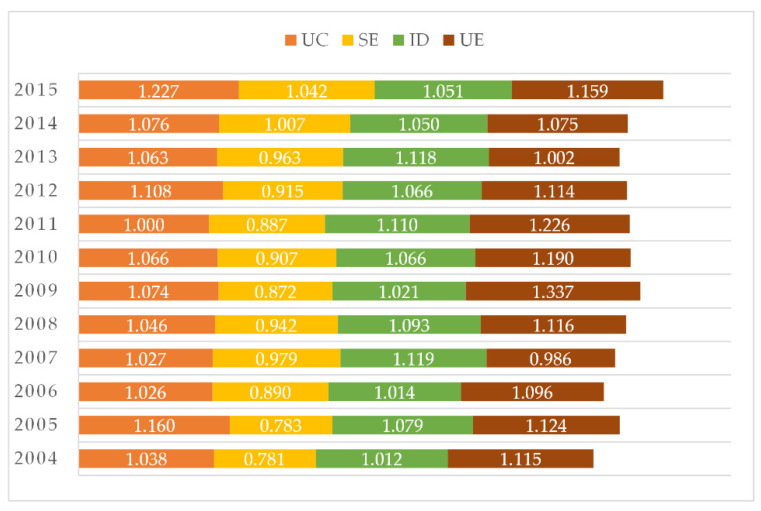
The four subsystems of the LCCC in the HCUA during 2004–2015.

From the point of view of the four subsystems, the sum of each subsystems of the HCUA reflects the contribution and change to the LCCC (Figure 4). Specifically, the SE subsystem has the largest increase, its value increasing from 0.781 in 2004 to 1.042 in 2015, and the contribution rate increased by 26.1%. The UE subsystem has the highest contribution with a value of 1.159 in 2015, surpassing the other three subsystems with low volatility. The contribution rate of ID is relatively stable within the time window.

### 4.2. Gradient Analysis of the LCCC for the HCUA

#### 4.2.1. Analysis for Four Gradients

**Figure 5:**
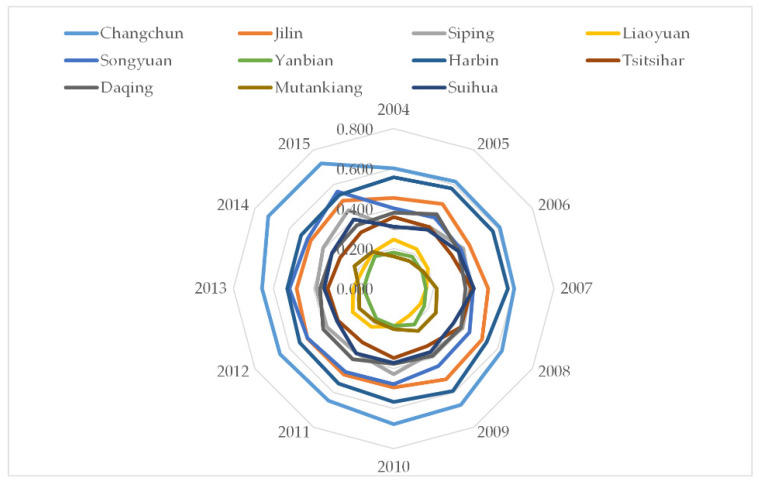
The LCCC of the cities in the HCUA during 2004–2015.

**Figure 6:**
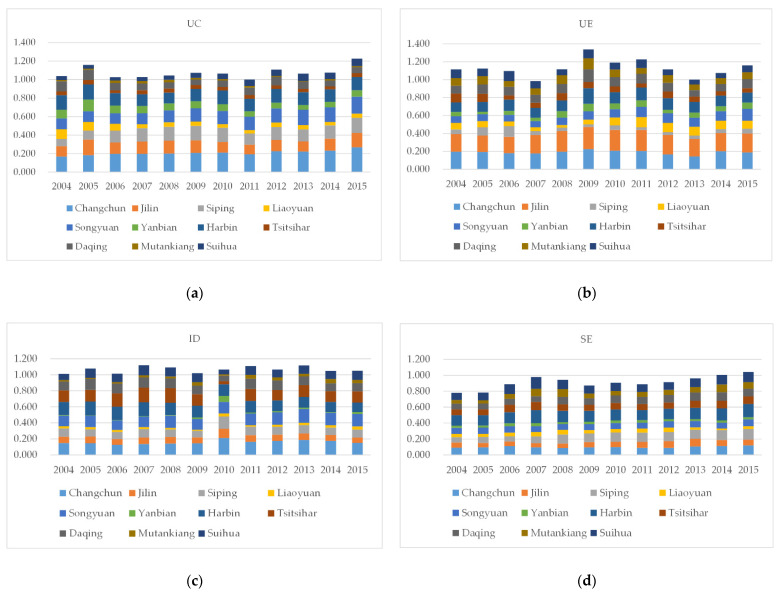
The LCCC of each subsystem of the cities in the HCUA. (**a**–**d**) are the LCCC of UC, UE, ID, and SE, respectively, during 2004–2015.

Figure 5 and Table 4, respectively, show the carrying capacity results of each city of the HCUA from 2004 to 2015, including the four gradients, namely excellent, good, inferior, and poor. Figure 6 illustrates each subsystem’s LCCC of the cities in the HCUA. The major findings are presented below.

For the first gradient (0.6–0.8), Changchun’s LCCC is significantly higher than the average value of the other cities of the HCUA, and the value increases from 0.602 to 0.723. Specifically, UE has an inverted U-shaped trend with the inflection point appearing in 2009, indicating that UE may be the obstacle for improving the land carrying capacity for the first gradient. Compared to other cities, the dynamics of each subsystem in Changchun is stable, which means the core city has location advantages and policy preferences. The level of rational allocation of urban resources is high.

For the second gradient (0.4–0.6), the included cities are Harbin, Jilin, and Songyuan. The secondary growth cities’ LCCC reveals the state of good carrying capacity. Harbin, one of the core cities, is higher than that of the other two cities, with an average of 0.556. In terms of subsystems, first, Harbin’s UE is significantly lower than Jilin, indicating the improvement of the material foundation and social economic accompanied by the consumption of resources and the environment. The linear development model of high consumption and emissions is still prevalent. Second, the urban land use efficiency of the second gradient needs to improve urgently. The ID subsystem of the three cities has a downward trend that will become the main factor for improving carrying capacity. Third, the UC subsystem has improved in recent years. Increasing road density has a stronger circulation capacity in the urban construction axis, which is conducive to the allocation of resources and economic development of the HCUA.

For the third gradient (0.2–0.4), which includes Siping, Daqing, Suihua, and Tsitsihar, these cities are at the inferior level. It is noteworthy that UE decreases as SE rises, and the development of the local economy is accompanied by the consumption and destruction of resources and the ecological environment, which is also the main reason for this state.

For the fourth gradient (0–0.2), the LCCC of Liaoyuan, Mutankiang, and Yanbian is already in the poor state. On the one hand, inefficient land use constrains the ID subsystem. On the other hand, these cities lack urban construction, whose infrastructure investment is low and road traffic is inadequate, resulting in low resource acquisition capacity. In contrast to the other three gradients, the UE subsystem trend is higher than the other three subsystems.

#### 4.2.2. Analysis of Dual-Core Cities

**Figure 7:**
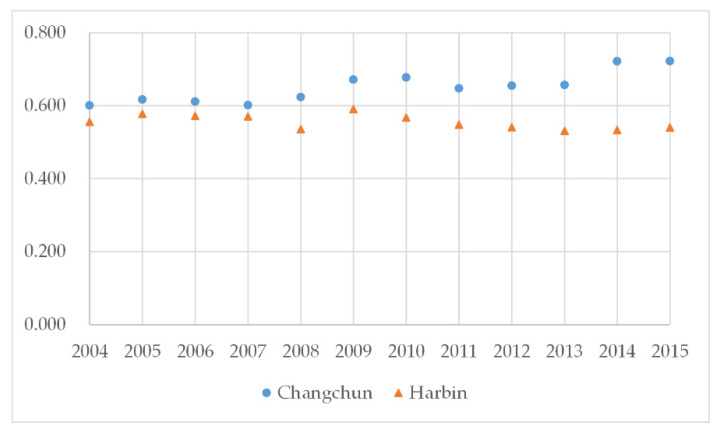
Characteristics of the LCCC in the core cities of the HCUA during 2004–2015.

As core cities of the HCUA, and with respect to the LCCC, the provincial capital cities of Changchun and Harbin are at the excellent and good levels, respectively. For Changchun, the average value of the LCCC is 0.651 during the period and it is greater than 0.6 every year, increasing after 2014 to 0.722. For Harbin, the average value of the LCCC is 0.556, which indicates a balanced state. In other words, the land resource of core cities can withstand more construction activities and a larger scale of development, while it is worth noting that Harbin’s LCCC has gradually declined since 2011. There are two main reasons. One is that the extensive economic growth model has damaged the urban ecological environment and deviated from the policy of sustainable development. The other is that the dual-core urban agglomeration model is likely to cause long-term competition and cooperation between core cities, which leads to a trend of reverse development (Figure 7).

### 4.3. Analysis of the Temporal and Spatial Differentiation of the LCCC for the HCUA

#### 4.3.1. Global Spatial Autocorrelation for LCCC

Global Moran’s I of LCCC was obtained using the spatial statistics module and vector data of Geoda software (Spatial Analysis Laboratory, Illinois, USA). The results show that Moran’s I > 0, but the *p*-value is less than 0.05 and the Z-score does not exceed the critical value of 1.65. The random characteristics of the data distribution trend indicate that the original hypothesis is not rejected. In summary, there is no obvious clustering characteristic of the LCCC of the HCUA in general.

#### 4.3.2. Local Autocorrelation Analysis for LCCC

**Figure 8:**
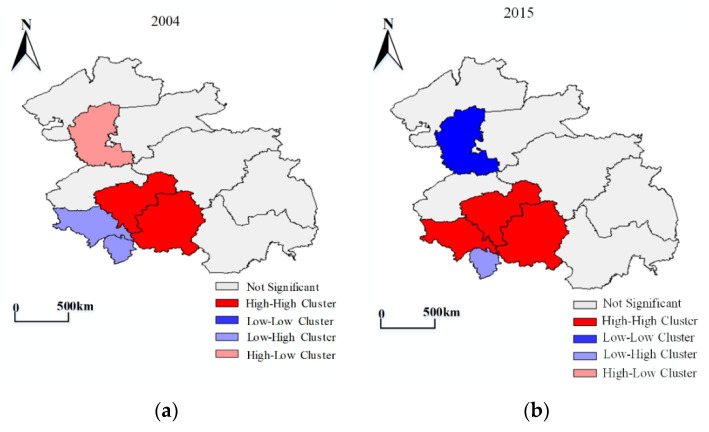
Spatial distribution of the LCCC in the HCUA. (**a**,**b**) are spatial distribution maps of LCCC in 2004 and 2015.

In order to further explore the spatial distribution characteristics of the LCCC for the HCUA, the cluster map function of the local Moran’s I module in Geoda software was used to obtain clustered and discrete distributions of 2004 and 2015, respectively. Figure 8 shows that the five cities have a similar spatial pattern of LCCC and are mainly distributed in the southern part of the HCUA. Changchun and Jilin are high–high clustering, which is a high value and its neighborhood. Liaoyuan is a cold spot with a low value and its neighborhood. The comparison of the time evolution of 2004 and 2015 shows that Siping developed from a cold spot to a hot spot, whereas Daqing changed from a high–low area to a cold spot area.

#### 4.3.3. Spatial Differentiation Evolution Analysis

**Figure 9:**
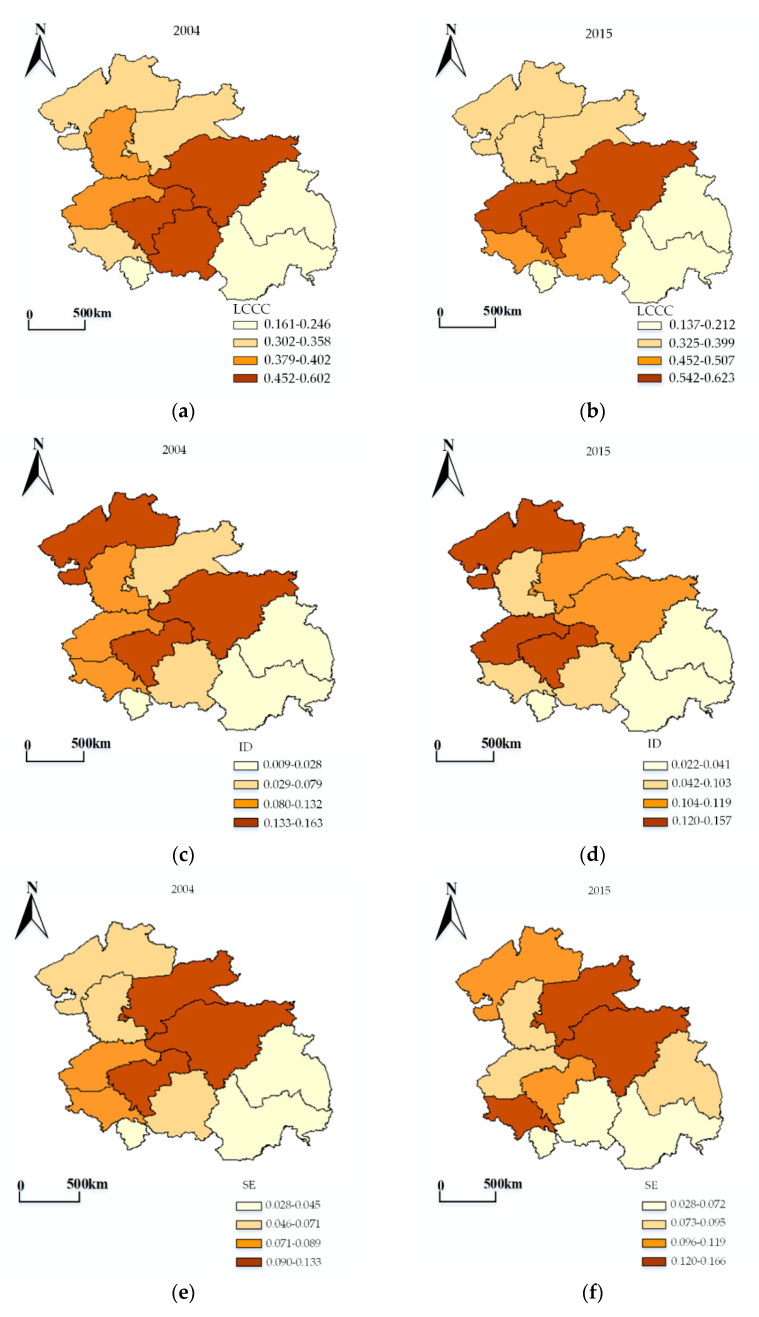

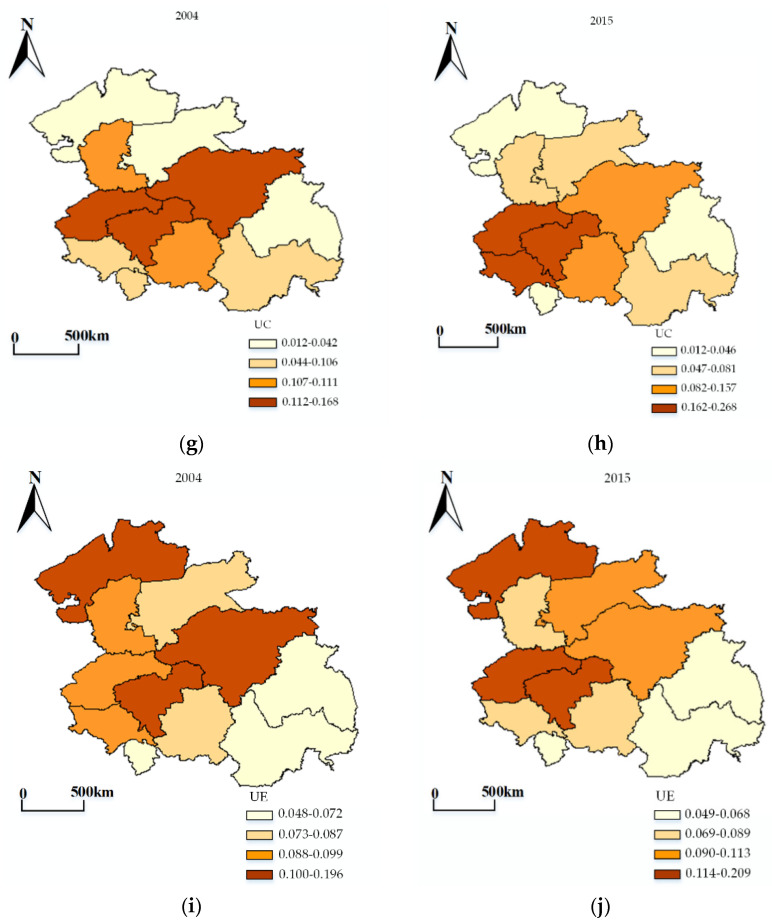
The temporal evolution and spatial differentiation of the LCCC in the HCUA. (**a**,**b**) are spatial and temporal differentiation maps of LCCC in 2004 and 2015. (**c**–**j**) are each subsystem’s spatial and temporal differentiation maps of LCCC in 2004 and 2015.

Figure 9 illustrates that the overall changes of LCCC are characterized by the spatial differentiation of fragmented agglomerations that “gradually decrease from the center to the periphery”. This “south high, north low” feature is similar to the economic and comprehensive benefits of land use in the northeastern region of China. Changchun and Harbin, which have the highest values, are the core areas of the cluster, followed by Jilin and Songyuan.

From the perspective of subsystem, some further results can be found. First, Harbin’s UC has declined one level in recent years. The cities around Changchun have seen some improvement. The higher areas are concentrated in the middle of the urban agglomeration. Second, the overall spatial distribution of SE has not changed much. Cities around the core, such as Jilin, Songyuan, Qiqihar, Suihua, and Siping, have upgraded, while there was a drop in Changchun’s grade. Third, higher UE is concentrated in the northern and central parts of the urban agglomerations, where clustering is more pronounced. UC and UE are evolving in opposite directions.

### 4.4. Discussion

In this study, the quantitative evaluation system of the LCCC provides a set of measurable indexes to guide scientific land planning and promote the efficiency of land use, enabling us to identify a sustainable land development model for urban agglomerations. From the perspective of the overall urban agglomeration, the LCCC of the HCUA is gradually improving, and we observe that benign urbanization and policy support are essential for ensuring regional sustainable development. UE is the factor that has the greatest influence on the LCCC, to which cities that develop and utilize land resources for economic development should give priority consideration. The constraints of urban ecological conditions should be considered in order to achieve a mutually reinforcing development state.

A previous study published in 2014 also focused on the LCCC of dual-core urban agglomeration [58]. This study found that the core cities in urban agglomerations have significantly higher carrying capacity than other cities, which is consistent with the basic characteristics of urban agglomeration development. In our study, from the perspective of each city of the HCUA, the gradients’ distribution of carrying capacity is unbalanced. The core cities Changchun and Harbin have greater development potential, which means that the core resources of the urban agglomeration are concentrated and reasonably distributed. The core area should be developed first and then the development should be radiated to the surrounding cities. This result is also consistent with the findings of scholars such as Peng [59] and Fang [60]. Additionally, according to Fang’s study on different structural urban agglomerations, it was concluded that the core cities of dual-core urban agglomerations tended to be consistent [61]. On the contrary, the finding of this study is that the LCCC of the core cities Changchun and Harbin shows a progressively opposite trend over time. This occurs because the structure of dual-core urban agglomerations has a certain degree of instability, which is prone to competition and monopoly in resource allocation between center cities, and the stability of dual-core urban agglomerations is lower than single-center urban agglomerations.

Some recent studies have focused mainly on the spatial evolution and distribution of land carrying capacity of urban agglomerations but have not considered the role of secondary growth poles in urban agglomerations without providing the driving factors to enhance the land carrying capacity [62]. Moreover, due to the obvious differentiation of urban land resources, it is the focus of spatial planning research to explore the differences in LCCC of core cities and secondary growth pole cities using different regions as evaluation units, and then to guide the spatial layout of land and economic development [63]. Therefore, in this study, Jilin and Songyuan were illustrated as the secondary growth pole cities. The HCUA is in the initial stage of development; driving the secondary growth pole cities through the development of Changchun and Harbin can increase the overall concentration of the HCUA. In addition, priority should be given to upgrading the industrial development subsystem of the secondary growth pole cities, which can provide a complementary and mutual promotion with the core cities, while enhancing the mobility of regional resources in the HCUA, so that the LCCC can be continuously improved.

## 5. Conclusions

This study aimed to establish an evaluation system for LCCC through the four dimensions of UC, SE, ID, and UE in the dynamic time window from 2004 to 2015, using a comprehensive index to calculate the LCCC of each city in the HCUA. This was carried out to explore the evolution and spatial differentiation of LCCC through spatial statistics and geographical distribution characteristics of urban agglomerations. The study framework can also be applied to other urban agglomerations with similar characteristics and provide a reference for policymakers. The main conclusions are as follows:

(1) The overall LCCC of the HCUA fluctuates moderately, showing a gradual upward trend from 3.945 in 2004 to 4.480 in 2015. This indicates that the sustainable land carrying capacity of the urban agglomeration is increasing due to the coordination of each subsystem. Among the four subsystems, UE is the main contributor to the overall integrated land carrying capacity. The SE subsystem is in a good state of development and the proportion of contribution has increased over the years. The spatial evolution of the four subsystems differs significantly, in which both UC and ID have a lower number of hot spot cities and lower clustering characteristics than SE and UE.
(2) The core cities of the HCUA, Changchun and Harbin, are in the states of excellent and good carrying capacity, respectively, which can withstand larger-scale development and construction. Four cities are at the inferior state, and three cities are poor. Moreover, the dual-core pattern is prone to long-term competition and cooperation, with a tendency of reverse development. The higher UC and ID in the safe carrying range make the land use structure of the region more stable, so that the LCCC utilization efficiency is in the rising stage, but the UE environment fluctuates greatly. The ID subsystem is the bottleneck to improve the LCCC for the cities at inferior and poor states.
(3) The spatial variation of the LCCC in the HCUA is large, which shows the characteristic of “gradually decreasing from the core city to the surrounding area”. Changchun, Jilin, Songyuan, Siping, and Daqing are characterized by the local spatial distribution of point-like dispersion and piece-like aggregation. Among them, Changchun and Jilin are high–high clustering areas, which drive Siping from a cold spot to a hot spot. Improving the land use efficiency of secondary growth pole cities has a positive effect on promoting the spatial sustainable development of the HCUA.

The results of the study help to deepen the understanding of the LCCC of the HCUA. They provide a basis for policymakers to take appropriate measures for different regions to better solve land problems. However, there are still some shortcomings in this study, as the index system does not take into account interregional liquidity factors. The spatio-temporal driving factor evolution of the LCCC has not been widely explored in depth, which requires further evaluation in future studies.

## Figures and Tables

**Table 1:** Land comprehensive carrying capacity (LCCC) indicator dimensions in existing research studies.

References	Indicator Dimension
[40]	Environment, Resource, Infrastructure, Ecological Civilization, Public Service
[41]	Resource supporting capacity, Ecological environment supporting capacity, Economic and technology supporting capacity, Social development supporting capacity
[18]	Resource, Eco-environment, Social resources, Economy, and Technology
[42]	Land social-developmental, Land ecological-environmental, Land economic-productive
[43]	Economic Resources, Environmental, Infrastructural, Transport
[44]	Economic, Social, Ecological/environmental/Spatial, Political, Resource

**Table 2:** Evaluation index system of LCCC.

Target Layer	Criterion Layer	Index Layer	Units	Description	Weight
Land comprehensive carrying capacity of the HCUA	Urban construction	Per capita housing area (+)	m^2^	Degree of urban land construction and scale of urban living space	0.121
		Road density (+)	km/km^2^	Development of construction land	0.055
		Per unit area infrastructure investment (+)	10,000 yuan/km^2^	Intensity of land input and output	0.119
	Social economy	Economic density (+)	10,000 yuan/km^2^	Output efficiency per unit area of land	0.092
		Engel’s Coefficient (−)	—	Level of living for residents	0.068
		Urbanization rate (−)	%	Scale of population gathering	0.055
	Industry development	Per unit area industrial output (+)	10,000 yuan/km^2^	Carrying efficiency and use intensity per unit of land	0.124
		Per capita grain output (+)	kilogram	Scale of land production	0.054
		Per capita farmland area (+)	ha	Support of regional land for industrial development	0.082
	Urban ecology	Domestic sewage treatment rate (+)	%	Ecological adaptive capacity of human activities	0.057
		Per capita public green area (+)	m^2^	Quality of the living environment	0.116
		Green coverage of urban area (+)	%	Natural condition of land resources	0.056

**Table 3:** Research data sources.

Data Types	Data Source
Socioeconomic data	Statistical Yearbook of Jilin and Heilongjiang Provinces (2004–2015)
	Statistical Bulletin of National Economic and Social Development (2004–2015)
Land use data	Resource and Environment Data cloud platform (www.resdc.cn)
Resource and environmental data	Environmental Statistics Yearbook (2004–2015)
	Environmental Status Bulletin (2004–2015)

**Table 4:** Classification standards of the LCCC in the HCUA.

Gradient	Level of LCCC	Cities
I	Excellent	Changchun
II	Good	Harbin, Jilin, Songyuan
III	Inferior	Siping, Daqing, Suihua, Tsitsihar
IV	Poor	Liaoyuan, Mutankiang, Yanbian

## Data Availability

The data presented in this study are available on request from the corresponding author.

## References

[1] Bertinelli L., Black D. (2004). Urbanization and growth. J. Urban Econ..

[2] Fang C. (2015). Scientifically selecting and hierarchically nurturing China’s urban agglomerations for the new normal. Bull. Chin. Acad. Sci..

[3] China State Council Home Page. http://www.gov.cn/zhengce/2014-03/16/content_2640075.htm.

[4] Su Y., Chen X., Liao J., Zhang H., Wang C., Ye Y., Wang Y. (2016). Modeling the optimal ecological security pattern for guiding the urban constructed land expansions. Urban For. Urban Green..

[5] Avelar S., Zah R., Tavares-Corrêa C. (2009). Linking socioeconomic classes and land cover data in Lima, Peru: Assessment through the application of remote sensing and GIS. Int. J. Appl. Earth Obs. Geoinf..

[6] Yuan Y., Chen D., Wu S., Mo L., Tong G., Yan D. (2019). Urban sprawl decreases the value of ecosystem services and intensifies the supply scarcity of ecosystem services in China. Sci. Total Environ..

[7] United Nations Home Page. https://www.un.org/development/desa/zh/news/population/world-population-prospects-2019.html.

[8] Gu C., Hu L., Cook I.G., Xu Y. (2017). China’s urbanization in 1949–2015: Processes and driving forces. Chin. Geogr. Sci..

[9] Wang J., Lin Y., Glendinning A., Xu Y. (2018). Land-use changes and land policies evolution in China’s urbanization processes. Land Use Policy.

[10] China State Council Home Page. http://www.gov.cn/zhengce/content/2011-06/08/content_1441.htm.

[11] Kessler J.J. (1994). Usefulness of the human carrying capacity concept in assessing ecological sustainability of land-use in semi-arid regions. Agric. Ecosyst. Environ..

[12] Onishi T. (1994). A capacity approach for sustainable urban development: An empirical study. Reg. Stud..

[13] Millington R., Gifford R. (1973). Energy and How We Live.

[14] Sun X., Qin J., Diao C., Zuo T. (2014). Spatial-Temporal Difference Analysis of Land Carrying Capacity Based on Man-Grain Relationship in Chongqing Municipality. J. Southwest Univ. Nat. Sci. Ed..

[15] Lane M. (2010). The carrying capacity imperative: Assessing regional carrying capacity methodologies for sustainable land-use planning. Land Use Policy.

[16] Yang J., Ding H. (2018). A quantitative assessment of sustainable development based on relative resource carrying capacity in Jiangsu province of China. Int. J. Environ. Res. Public Health.

[17] Ma P., Ye G., Peng X., Liu J., Qi J., Jia S. (2017). Development of an index system for evaluation of ecological carrying capacity of marine ecosystems. Ocean Coast. Manag..

[18] Cui G., Zhang X., Zhang Z., Cao Y., Liu X. (2019). Comprehensive land carrying capacities of the Cities in the Shandong Peninsula blue economic zone and their spatio-temporal variations. Sustainability.

[19] Wei C., Guo Z.Y., Wu J.P., Ye S.F. (2014). Constructing an assessment indices system to analyze integrated regional carrying capacity in the coastal zones—A case in Nantong. Ocean Coast. Manag..

[20] Cheng J., Zhou K., Chen D., Fan J. (2016). Evaluation and analysis of provincial differences in resources and environment carrying capacity in China. Chin. Geogr. Sci..

[21] Huang Q., Wang R., Ren Z., Li J., Zhang H. (2007). Regional ecological security assessment based on long periods of ecological footprint analysis. Resour. Conserv. Recycl..

[22] Zeev S., Meidad K., Avinoam M. (2014). A multi-spatial scale approach to urban sustainability—An illustration of the domestic and global hinterlands of the city of Beer-Sheva. Land Use Policy.

[23] Dai D., Sun M., Lv X., Lei K. (2020). Evaluating water resource sustainability from the perspective of water resource carrying capacity, a case study of the Yongding River watershed in Beijing-Tianjin-Hebei region, China. Environ. Sci. Pollut. Res..

[24] Yang J., Lei K., Khu S., Meng W. (2014). Assessment of Water Resources Carrying Capacity for Sustainable Development Based on a System Dynamics Model: A Case Study of Tieling City, China. Water Resour. Manag..

[25] Li K., Jin X., Ma D., Jiang P. (2019). Evaluation of resource and environmental carrying capacity of China’s rapid-urbanization areas-A case study of Xinbei District, Changzhou. Land.

[26] Nakajima E.S., Ortega E. (2016). Carrying capacity using emergy and a new calculation of the ecological footprint. Ecol. Indic..

[27] Qian Y., Tang L., Qiu Q., Xu T., Liao J. (2015). A comparative analysis on assessment of land carrying capacity with ecological footprint analysis and index system method. PLoS ONE.

[28] Chen C.H., Liu W.L., Liaw S.L., Yu C.H. (2005). Development of a dynamic strategy planning theory and system for sustainable river basin land use management. Sci. Total Environ..

[29] Guneralp B., Reilly M.K., Seto K.C. (2012). Capturing multiscalar feedbacks in urban land change: A coupled system dynamics spatial logistic approach. Environ. Plan. B Plan. Des..

[30] Aspinall R., Staiano M. (2017). A conceptual model for land system dynamics as a coupled human-environment system. Land.

[31] Ren C., Guo P., Li M., Li R. (2016). An innovative method for water resources carrying capacity research—Metabolic theory of regional water resources. J. Environ. Manag..

[32] Widodo B., Lupyanto R., Sulistiono B., Harjito D.A., Hamidin J., Hapsari E., Yasin M., Ellinda C. (2015). Analysis of Environmental Carrying Capacity for the Development of Sustainable Settlement in Yogyakarta Urban Area. Procedia Environ. Sci..

[33] Huang Y., Li L., Yu Y. (2018). Do urban agglomerations outperform non-agglomerations? A new perspective on exploring the eco-efficiency of Yangtze River Economic Belt in China. J. Clean. Prod..

[34] Liu H. (2012). Comprehensive carrying capacity of the urban agglomeration in the Yangtze River Delta, China. Habitat Int..

[35] Li Z.T., Li M., Xia B.C. (2020). Spatio-temporal dynamics of ecological security pattern of the Pearl River Delta urban agglomeration based on LUCC simulation. Ecol. Indic..

[36] Wang Z. (2018). Land spatial development based on carrying capacity, land development potential, and efficiency of urban agglomerations in China. Sustainability.

[37] Pahuluan A., Soeprobowati T.R., Hadiyanto H. (2017). Environmental carrying capacity based on land balance for evaluation planning of spatial and regional in Solok regency, West Sumatra. J. Ecol. Eng..

[38] Shi Y., Wang H., Yin C. (2013). Evaluation method of urban land population carrying capacity based on GIS-A case of Shanghai, China. Comput. Environ. Urban Syst..

[39] Comber A., Wadsworth R., Fisher P. (2008). Using semantics to clarify the conceptual confusion between land cover and land use: The example of ‘forest’. J. Land Use Sci..

[40] Su Y., Xue H., Liang H. (2019). An evaluation model for urban comprehensive carrying capacity: An empirical case from harbin city. Int. J. Environ. Res. Public Health.

[41] Xue Q., Yang X., Wu F. (2020). A three-stage hybrid model for the regional assessment, spatial pattern analysis and source apportionment of the land resources comprehensive supporting capacity in the Yangtze River Delta urban agglomeration. Sci. Total Environ..

[42] Yang Y., Meng G. (2019). A bibliometric analysis of comparative research on the evolution of international and Chinese ecological footprint research hotspots and frontiers since 2000. Ecol. Indic..

[43] Wei Y., Huang C., Li J., Xie L. (2016). An evaluation model for urban carrying capacity: A case study of China’s mega-cities. Habitat Int..

[44] Bonsu N.O., Dhubháin Á.N., O’Connor D. (2017). Evaluating the use of an integrated forest land-use planning approach in addressing forest ecosystem services confliciting demands: Expereince within an Irish forest landscape. Futures.

[45] Ministry of Natural Resources of the People’s Republic of China Home Page. http://www.mnr.gov.cn/.

[46] Li C., Gao X., He B.J., Wu J., Wu K. (2019). Coupling coordination relationships between urban-industrial land use efficiency and accessibility of highway networks: Evidence from Beijing-Tianjin-Hebei Urban Agglomeration, China. Sustainability.

[47] Liao S., Wu Y., Wong S.W., Shen L. (2020). Provincial perspective analysis on the coordination between urbanization growth and resource environment carrying capacity (RECC) in China. Sci. Total Environ..

[48] Haase D., Frantzeskaki N., Elmqvist T. (2014). Ecosystem services in urban landscapes: Practical applications and governance implications. Ambio.

[49] Xie H., Zhu Z., Wang B., Liu G., Zhai Q. (2018). Does the expansion of urban construction land promote regional economic growth in China? Evidence from 108 cities in the Yangtze River economic belt. Sustainability.

[50] Fu J.Y., Wu Y., Zang C.F., Zhang C.M. (2020). Economic and resource and environment carrying capacity trade-off analysis in the Haihe River basin in China. J. Clean. Prod..

[51] Niedertscheider M., Kuemmerle T., Müller D., Erb K.H. (2014). Exploring the effects of drastic institutional and socio-economic changes on land system dynamics in Germany between 1883 and 2007. Glob. Environ. Chang..

[52] Tang L., Wang D. (2018). Optimization of county-level land resource allocation through the improvement of allocation efficiency from the perspective of sustainable development. Int. J. Environ. Res. Public Health.

[53] Yang N., Li J., Lu B., Luo M., Li L. (2019). Exploring the spatial pattern and influencing factors of land carrying capacity in Wuhan. Sustainability.

[54] Zhou D., Lin Z., Lim S.H. (2019). Spatial characteristics and risk factor identification for land use spatial conflicts in a rapid urbanization region in China. Environ. Monit. Assess..

[55] Wu X., Hu F. (2020). Analysis of ecological carrying capacity using a fuzzy comprehensive evaluation method. Ecol. Indic..

[56] Anselin L. (1988). Spatial Economitrics: Methods and Models.

[57] Laura K. (2017). The road to a market-oriented monetary policy and the ‘new moral’ monetary policy regime in China. Financ. Econ. Rev..

[58] Fang C. (2019). The basic law of the formation and expansion in urban agglomerations. J. Geogr. Sci..

[59] Peng B., Wang Y., Elahi E., Wei G. (2018). Evaluation and prediction of the ecological footprint and ecological carrying capacity for yangtze river urban agglomeration based on the grey model. Int. J. Environ. Res. Public Health.

[60] Fang C., Yu D. (2017). Urban agglomeration: An evolving concept of an emerging phenomenon. Landsc. Urban Plan..

[61] Dai E., Wu Z., Du X. (2018). A gradient analysis on urban sprawl and urban landscape pattern between 1985 and 2000 in the Pearl River Delta, China. Front. Earth Sci..

[62] Jia X., Shao M., Yu D., Zhang Y., Binley A. (2019). Spatial variations in soil-water carrying capacity of three typical revegetation species on the Loess Plateau, China. Agric. Ecosyst. Environ..

[63] Zhou X., Luo R., An Q., Wang S., Lev B. (2019). Water resource environmental carrying capacity-based reward and penalty mechanism: A DEA benchmarking approach. J. Clean. Prod..

